# Prevalence of fibromyalgia in Egyptian patients with systemic lupus erythematosus and its impact on health-related quality of life

**DOI:** 10.1186/s42358-026-00571-x

**Published:** 2026-08-24

**Authors:** Samar Tharwat, Rahma A. Elziaty, Fatma Hamdy, Abdelrahman Mohamed Elsayed, Esraa A. Talaat, Enas S. Zahran

**Affiliations:** 1 Rheumatology & Immunology Unit, Department of Internal Medicine, Faculty of Medicine, Mansoura University, Mansoura University Hospital, El Gomhouria St, Mansoura, Dakahlia Governorate 35511 Egypt; 2 Department of Internal Medicine, Faculty of Medicine, Horus University, New Damietta, Egypt; 3 Internal Medicine Department, Rheumatology Division, Faculty of Medicine, Ain Shams University, Cairo, Egypt; 4 Faculty of Medicine, Mansoura University, Mansoura, Egypt; 5 Department of Rheumatology, Rehabilitation and Physical Medicine, Faculty of Medicine, Assiut University, Assiut, Egypt; 6 Department of Internal Medicine, Rheumatology & Immunology, Faculty of Medicine, Menoufia University, Shebin El-kom, Egypt

**Keywords:** Fibromyalgia, Systemic lupus erythematosus quality of life, Egypt, Sleep, SF36

## Abstract

**Background:**

Fibromyalgia (FM) is a rheumatic disorder characterized by widespread persistent pain, hyperalgesia, and allodynia.

**Objectives:**

The aim of this study was to assess the prevalence of FM among Egyptian patients with systemic lupus erythematosus (SLE) and its effect on their health-related quality of life (HRQoL).

**Materials and methods:**

This cross-sectional analytical multicenter study was conducted on 550 Egyptian SLE patients. The patients’ sociodemographic characteristics, clinical and therapeutic data, and sleeping patterns were collected. SLE disease activity index (SLEDAI) was assessed. We employed the 2016 revisions to the 2010/2011 FM diagnostic criteria to determine the presence of FM and the patients administered the Short-Form Health Survey-36 (SF-36).

**Results:**

The mean (SD) age was 32.72 (9.90) years, and 91.3% were female. Of 550 Egyptian SLE patients, 119 (21.6%) fulfilled the diagnostic criteria of FM. The FM group was statistically significantly associated with higher age (*P* = 0.001), body mass index (BMI) (*P* = 0.008), longer SLE duration (*p* < 0.001), and higher scores of SLEDAI (*p* < 0.001). Also, living alone (*p* < 0.001), chronic psychiatric disease (*p* = 0.008), chronic kidney disease (*p* = 0.025), and chronic hemodialysis (*p* = 0.042) were significantly higher among the FM group. Sleep disorders, such as snoring and sleep apnea, were more prevalent among FM patients. The average sleep hours were significantly lower among the FM group (*p* < 0.001). FM patients had statistically significantly lower scores across all domains of SF36.

**Conclusion:**

FM is prevalent among Egyptian SLE patients, and shows significant associations *with*age, BMI, SLE duration and activity, and disturbed sleep pattern and has a negative impact on their HRQoL. Therefore, early detection and treatment are recommended.

## Key points

The prevalence of FM in Egyptian SLE patients is significant.

FM impacts HRQoL in SLE patients.

This holistic approach is essential for fostering better long-term health outcomes in SLE patients.

## Introduction

Systemic lupus erythematosus (SLE) is a complex and challenging disease, characterized by systemic and organ-specific clinical manifestations [1]. The disease has a waxing and waning course and may be associated with life-threatening complications [2]. The significant morbidity, prolonged illness course, and excessive reliance on corticosteroid treatment all contribute to long-term organ damage [3]. The diagnosis of SLE relies on distinctive clinical manifestations affecting the skin, joints, kidneys, and central nervous system, in addition to serological parameters such as antinuclear antibodies (ANA) [2].

Musculoskeletal (MSK) involvement is one of the most prevalent features of SLE and may significantly impact patients’ general well-being and health-related quality of life (HRQoL) [4]. The inflammatory MSK manifestations of SLE can exhibit various phenotypes, displaying distinct clinical characteristics that may vary among individuals. The phenotypes, ranging from mild arthralgia to severe erosive arthritis, contribute to the variability of arthritis presentations in SLE patients [5]. Nonetheless, noninflammatory MSK involvement such as FM may occur in patients with SLE, exacerbating the disease burden [6].

Fibromyalgia (FM) is a rheumatic disorder characterized by widespread persistent pain, hyperalgesia, and allodynia. Fatigue, sleep difficulties, morning stiffness, headache, and paresthesia are frequently observed symptoms [7]. FM impairs mental, social, and physical functioning, characterized primarily by widespread MSK pain and increased pain sensitivity [8, 9].

Health-related quality of life (HRQoL) is a subjective assessment that takes into account each person’s particular perspective, experiences, and goals [11]. HRQoL denotes a comprehensive understanding of overall functional health [12], encompassing aspects such as personal health satisfaction, along with physical, psychological, and socio-economic well-being [13]. For patients with chronic illnesses, HRQoL has emerged as an essential indicator for comprehensive and integrated care [14].

FM among Egyptian patients with SLE and its effect on their HRQoL are not deeply studied. So, the aim of this study was to assess the prevalence of FM among Egyptian patients with SLE and its effect on their HRQoL.

## Patients and methods

### Study design and setting

This cross-sectional analytical multicenter study was conducted on 550 Egyptian SLE patients between March and August 2025. We collected the patients from tertiary rheumatology centers, both inpatient and outpatient, across multiple governorates in Egypt, including Dakahlia, Gharbia, Cairo, Assiut, and Menoufia. The study was reported in accordance with the Strengthening the Reporting of Observational Studies in Epidemiology (STROBE) guidelines for cross-sectional studies. The inclusion criteria were the following: (a) age > 18 years; (b) fulfilling the 2012 Systemic Lupus International Collaborating Clinics (SLICC) classification criteria for SLE [15]. The study included all eligible patients. We excluded all patients diagnosed with other autoimmune or rheumatic diseases from the outset. We also excluded patients with chronic debilitating diseases, like advanced liver cirrhosis, heart failure, and metastatic malignancy. Additionally, patients with a history of major trauma, orthopedic surgery, joint replacement, or a recent history of intramuscular or intraarticular steroid injections were excluded.

**Fig. 1:**
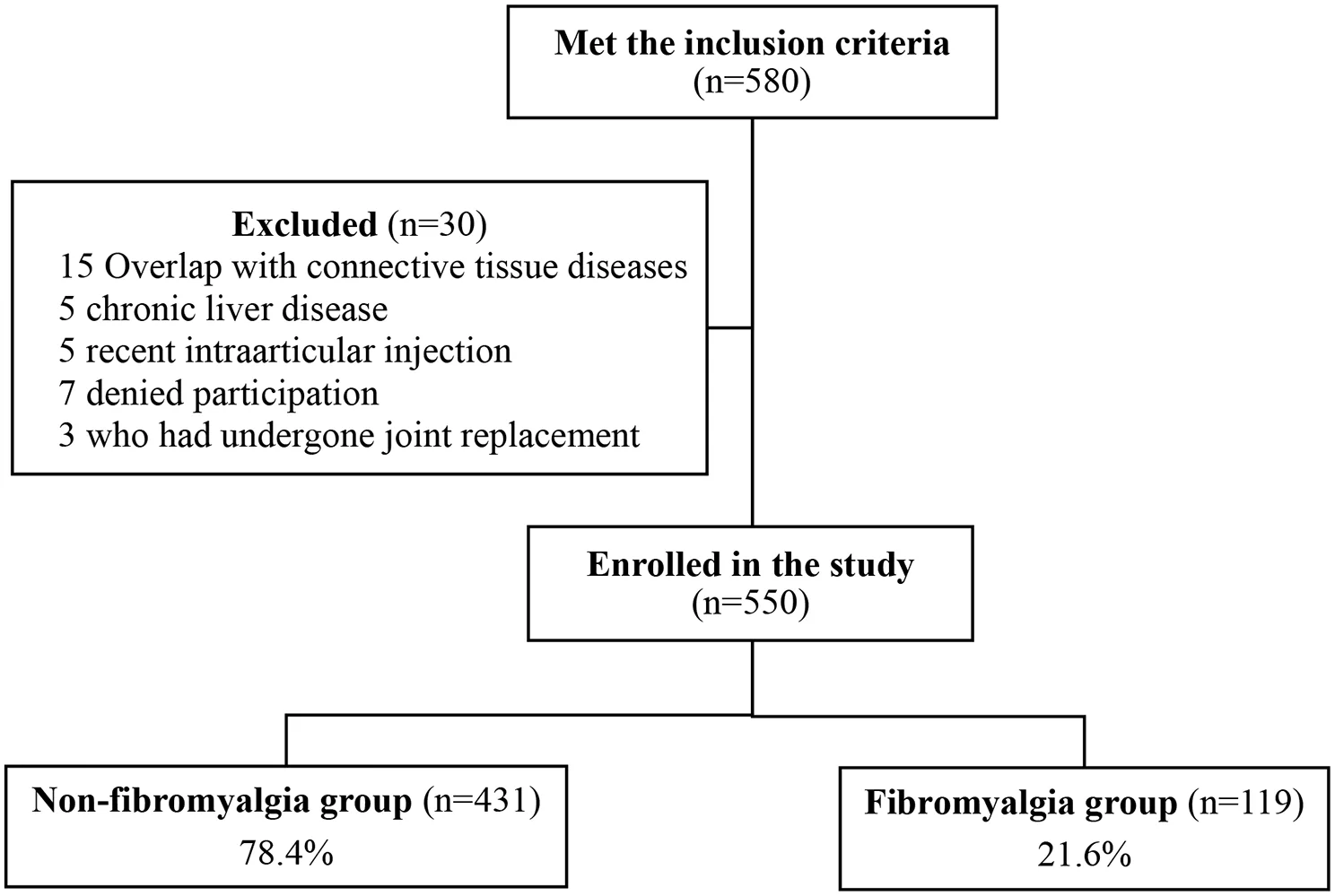
Flowchart of the study

Five hundred eighty consecutive patients met the inclusion criteria and were initially enrolled in this study. We excluded 30 patients: 15 with overlapping connective tissue diseases, 5 with chronic liver disease, 5 with recent intraarticular injection, 7 who denied participation, and 3 who had undergone joint replacement. Ultimately, 550 patients were incorporated into the study. Figure 1 presents a flow chart depicting the selection of participants in the study.

### Sample size calculation

The required sample size was estimated a priori using a two-proportion z-test based on the expected prevalence of FM in SLE patients (15.8–22.1%) in SLE cohorts, a conservative expected prevalence of 20% was adopted. Assuming a margin of error of 3.5% (precision level), a two-sided significance level of α = 0.05 (Z α/2 = 1.96), and a power of 80% (Zβ = 0.842), the minimum required sample size was calculated using the formula n = Z²α/2 × P(1 − P) / d², where P represents the anticipated prevalence and d denotes the acceptable margin of error, yielding ≈ 504 patients. Accounting for an anticipated non-response or exclusion rate of approximately 10%, the adjusted target sample size was inflated to approximately 560 patients. Ultimately, 580 consecutive eligible patients were screened, of whom 550 met all inclusion criteria and were enrolled, thereby satisfying and exceeding the calculated minimum sample size requirement and ensuring adequate statistical power for the primary and secondary analyses.

### Ethical consideration

This study adhered to the principles of the Helsinki Declaration [16], and the study received approval from the Institutional Research Board of the Faculty of Medicine at Mansoura University (approval registration number: R.24.12.2931). All participants were apprised of the research aims, scope, and their rights. Informed written consent was obtained from all participants.

### Baseline sociodemographic characteristics

The patients’ sociodemographic data were obtained from the patients and included age, marital status, education level, residence, employment status, life habits, and level of physical activity. We evaluated each patient’s weight and height and calculated their body mass index (BMI).

### Clinical and therapeutic data

We extracted clinical and therapeutic data from the patients’ computerized and written medical records. Clinical data included age at SLE diagnosis, disease duration, and associated comorbidities. Therapeutic data included corticosteroids, antimalarials, azathioprine, methotrexate, mycophenolate mofetil, and biologics.

The patients were evaluated for the presence of other relevant manifestations such as fatigue, irritable bowel syndrome, amnesia, hypothyroidism, headache, anxiety, depressed mood, low back pain (LBP). History of traffic accidents and family history of FM were obtained and reported. Information regarding chronic psychiatric disease was obtained from patients’ documented medical records and recorded as a physician-diagnated chronic psychiatric disorder (yes/no).A thorough clinical assessment was done by an expert rheumatologist, and the SLE disease activity index (SLEDAI) [17] was determined.

### Sleeping pattern

The patients were questioned regarding their sleeping habits, such as when they slept and how many hours, they typically slept each day. They were also asked if they snore when they sleep, take sleeping drugs, wake up throughout the night, or have any other sleeping issues.

### Diagnosis of fibromyalgia

The study was originally conducted to address inquiries regarding the existence of FM among SLE patients. The assessment for the existence of FM was conducted using the 2016 revisions [18] to the 2010/2011 FM diagnostic criteria, which involved evaluating the widespread pain index (WPI) and the symptom severity score (SSS). Participants were classified as positive for FM when all of the following criteria were met: (a) WPI score of 7 and SSS of 5, or WPI score of 4–6 and SSS of 9, or WPI of 4–6 and SSS score of 9,(b) Generalized pain was characterized as pain occurring in a minimum of four body areas, (c)Symptoms had persisted for a minimum of 3 months at a comparable intensity.

### Short-form health survey-36 (SF-36)

The HRQoL was evaluated using the SF-36. The SF-36 [19] comprises a multi-item measure that assesses eight health domains: physical function (PF), social function (SF), role function–physical (RFP), role function–emotional (RFE), emotional well-being (EW), vitality (VT), bodily pain (BP), and general health perception (GHP). Each domain spans from 0 to 100, with higher scores indicating better quality of life. Furthermore, the SF-36 includes two subscales: the physical component summary (PCS) and the mental component summary (MCS).

### Statistical analysis

The Statistical Package for Social Science (SPSS) version 22 software was used to analyze the data. The distribution of continuous variables was assessed for normality using the Shapiro-Wilk test. Normally distributed continuous variables were summarized as mean ± standard deviation (SD), while non-normally distributed variables were presented as median (minimum-maximum). Qualitative data were presented as frequencies and percentages. For comparisons between the FM and non-FM, the independent samples t-test was used for normally distributed continuous variables, and the Mann-Whitney U test was applied for non-normally distributed variables. We utilized the Chi-square test or the Fisher exact test, as appropriate, to compare qualitative variables. Univariate logistic regression analysis was performed to identify factors associated with fibromyalgia, with each variable entered individually into the model. Variables with a p-value < 0.05 in the univariate analysis were considered candidates for the multivariate logistic regression analysis. All enrolled patients completed the full study protocol, and there were no missing data for any of the variables analyzed. A p-value below 0.05 was deemed statistically significant.

## Results

This cross-sectional observational study included 550 SLE patients. The mean (SD) age was 32.72 (9.90) years, and most of them were female (91.3%) and married (63.5%). Only 4.5% were living alone. About 70% reported a moderate level of physical activity. One hundred and nineteen participants (21.6%) fulfilled the diagnostic criteria of FM. Table 1 illustrated the baseline demographic characteristics of those with and without FM. By comparing the two groups, we found that those with FM had a statistically significantly higher age (*P* = 0.001), weight (*P* = 0.003), and BMI (*P* = 0.008). Additionally, FM was more prevalent in those who were living alone (*p* < 0.001). The level of physical activity was statistically significantly lower in the FM group (*p* < 0.001). On the other hand, there was no statistically significant difference between the two groups regarding gender, marital status, and socioeconomic status.

**Table 1:** Baseline demographic characteristics of the study SLE patients (*n* = 550)

Variable mean ± SD, *n* (%), median(min.-max.)	Total (*n* = 550)	Non-fibromyalgia group (*n* = 431) 78.4%	Fibromyalgia group (*n* = 119) 21.6%	*P*
Age, years	32.72 ± 9.90	32.02 ± 9.97	35.26 ± 9.25	**0.001**
Gender				
Male Female	48 (8.7) 502 (91.3)	41 (9.5) 390 (90.5)	7 (5.9) 112 (94.1)	0.214
Marital status				
Single Married Widow Divorced	158 (28.7) 349 (63.5) 18 (3.3) 25 (4.5)	131 (30.4) 270 (62.6) 10 (2.3) 20 (4.6)	27 (22.7) 79 (66.4) 8 (6.7) 5 (4.2)	**0.053**
Living alone	25 (4.5)	12 (2.8	13 (10.9)	**< 0.001**
Education level				
Not educated Primary school Secondary school High school Graduate Postgraduate	54 (9.8) 9 (1.6) 43 (7.8) 217 (39.5) 192 (34.9) 35 (6.4)	44 (10.2) 7 (1.6) 34 (7.9) 166 (38.5) 157 (36.4) 23 (5.3)	10 (8.4) 2 (1.7) 9 (7.6) 51 (42.9) 35 (29.4) 12 (10.1)	0.370
Employment status				
Not employed Employed Retired Student	329 (59.8) 157 (28.5) 3 (0.5) 61 (11.1)	254 (58.9) 122 (28.3) 1 (0.2) 54 (12.5)	75 (63.0) 35 (29.4) 2 (1.7) 7 (5.9)	**0.055**
Residence				
Urban Rural	286 (52.0) 264 (48.0)	224 (52.0) 207 (48.0)	62 (52.1) 57 (47.9)	0.980
Socioeconomic status				
Low Moderate High	141 (25.6) 397 (72.2) 12 (2.2)	111 (25.8) 311 (72.2) 9 (2.1)	30 (25.2) 86 (72.3) 3 (2.5)	0.956
Life habits				
Smoking Alcohol consumption Exercise practice	35 (6.4) 4 (0.7) 41 (7.5)	26 (6.0) 2 (0.5) 32 (7.4)	9 (7.6) 2 (1.7) 9 (7.6)	0.545 **0.206** **0.959**
Physical activity level				
Low Moderate High	146 (26.5) 389 (70.7) 15 (2.7)	93 (21.6) 327 (75.9) 11 (2.6)	53 (44.5) 62 (52.1) 4 (3.4)	**< 0.001**
Treatment payment system				
Health insurance State expense Patient expense	123 (22.4) 284 (51.6) 143 (26.0)	106 (24.6) 225 (52.2) 100 (23.2)	17 (14.3) 59 (49.6) 43 (36.1)	**0.005**
Anthropometric measures				
Weight, Kg	71.90 ± 14.23	70.89 ± 13.71	75.58 ± 15.48	**0.003**
Height, m	1.62 ± 0.07	1.62±0.07	1.63±0.062	0.398
Body mass index, Kg/m^2^	27.34 ± 5.67	27.00 ± 5.67	28.54 ± 5.55	**0.008**

Table 2 displays the clinical and therapeutic data for the study SLE patients, along with the differences between FM and non-FM groups. There was a statistically significant difference between the two groups regarding age at SLE diagnosis (*p* < 0.001). SLEDAI scores were significantly higher in the FM group compared to the non-FM group (*p* < 0.001). The prevalence of chronic kidney disease (*p* = 0.025), chronic hemodialysis (*p* = 0.042), chronic psychiatric disease (*p* = 0.008), hypertension (*p* = 0.044), and diabetes mellitus (*p* = 0.001) was higher among FM patients. Other associated manifestations like fatigue (*p* < 0.001), sleep disturbance (*p* < 0.001), headache (*p* < 0.001), anxious mood (*p* < 0.001), depressed mood (*p* < 0.001), and low back pain (*p* < 0.001) were statistically significantly higher in the FM group. Not only that, but the frequency of traffic accidents and MTX administration was substantially higher in the FM group (*p* = 0.002,0.001, respectively).

**Table 2:** Clinical and therapeutic data of the study SLE patients (*n* = 550)

Variable mean ± SD, *n* (%), median(min.-max.)	Total (*n* = 550)	Non-fibromyalgia group (*n* = 431)	Fibromyalgia group (*n* = 119)	*P*
Clinical SLE data				
Age at SLE diagnosis, years	27.10 ± 9.08	26.39 ± 9.02	29.69 ± 8.86	**< 0.001**
Diseases duration, months	48 (1-420)	48 (1-420)	48 (2-228)	0.499
SLE diseases activity index				
No Mild Moderate Sever	40 (7.3) 176 (32.0) 252 (45.8) 82 (14.9)	39 (9.0) 159 (36.9) 189 (43.9 44 (10.2)	1 (0.8) 17 (14.3) 63 (52.9) 38 (31.9)	**< 0.001**
Family history for fibromyalgia	46 (8.4	37 (8.6	9 (7.6)	0.722
Associated comorbidities				
Lupus nephritis	227 (41.3)	179 (41.5)	48 (40.3)	0.815
Chronic kidney disease	92 (16.7)	64 (14.8)	28 (23.5)	**0.025**
Chronic hemodialysis	20 (3.6	12 (2.8)	8 (6.7)	**0.042**
Renal transplant	1 (0.2)	1 (0.2)	-	1.00
Chronic psychiatric disease	46 (8.4)	29 (6.7	17 (14.3)	**0.008**
Seizures	76 (13.8)	55 (12.8	21 (17.6)	0.172
Stroke	43 (7.8)	30 (7.0)	13 (10.9)	0.154
Chronic heart disease	36 (6.5)	23 (5.3)	13 (10.9)	**0.029**
Chronic lung disease	45 (8.2)	25 (5.8)	20 (16.8)	**< 0.001**
Hypertension	139 (25.3)	95 (22.0)	44 (37.0)	**0.044**
Diabetes mellitus	56 (10.2)	38 (8.8)	18 (15.1)	**0.001**
Associated manifestations				
Fatigue	268 (48.7)	178 (41.3)	90 (75.6)	**< 0.001**
Irritable bowel syndrome	287 (52.2)	191 (44.3)	96 (80.7)	**< 0.001**
Amnesia	310 (56.4)	211 (49.0)	99 (83.2)	**< 0.001**
Hypothyroidism	70 (12.7)	51 (11.8)	19 (16.0)	0.231
Headache	352 (64.0)	244 (56.6)	108 (90.8)	**< 0.001**
Anxious mood	327 (59.5)	219 (50.8)	108 (90.8)	**< 0.001**
Depressed mood	220 (40.0)	140 (32.5)	80 (67.2)	**< 0.001**
Low back pain	276 (50.2)	170 (39.4)	106 (89.1)	**< 0.001**
Traffic accident	26 (4.7)	14 (3.2)	12 (10.1)	**0.002**
Therapeutic data				
Corticosteroids	484 (88.0)	376 (87.2)	108 (90.8)	0.296
Antimalarials	495 (90.0)	386 (89.6)	109 (91.6)	0.512
Azathioprine	321 (58.4)	249 (57.8)	72 (60.5)	0.593
Methotrexate	71 (12.9)	45 (10.4)	26 (21.8)	**0.001**
Mycophenolate	233 (42.4)	187 (43.4)	46 (38.7)	0.355
Biologics	43 (7.8)	32 (7.4)	11 (9.2)	0.513

**Fig. 2:**
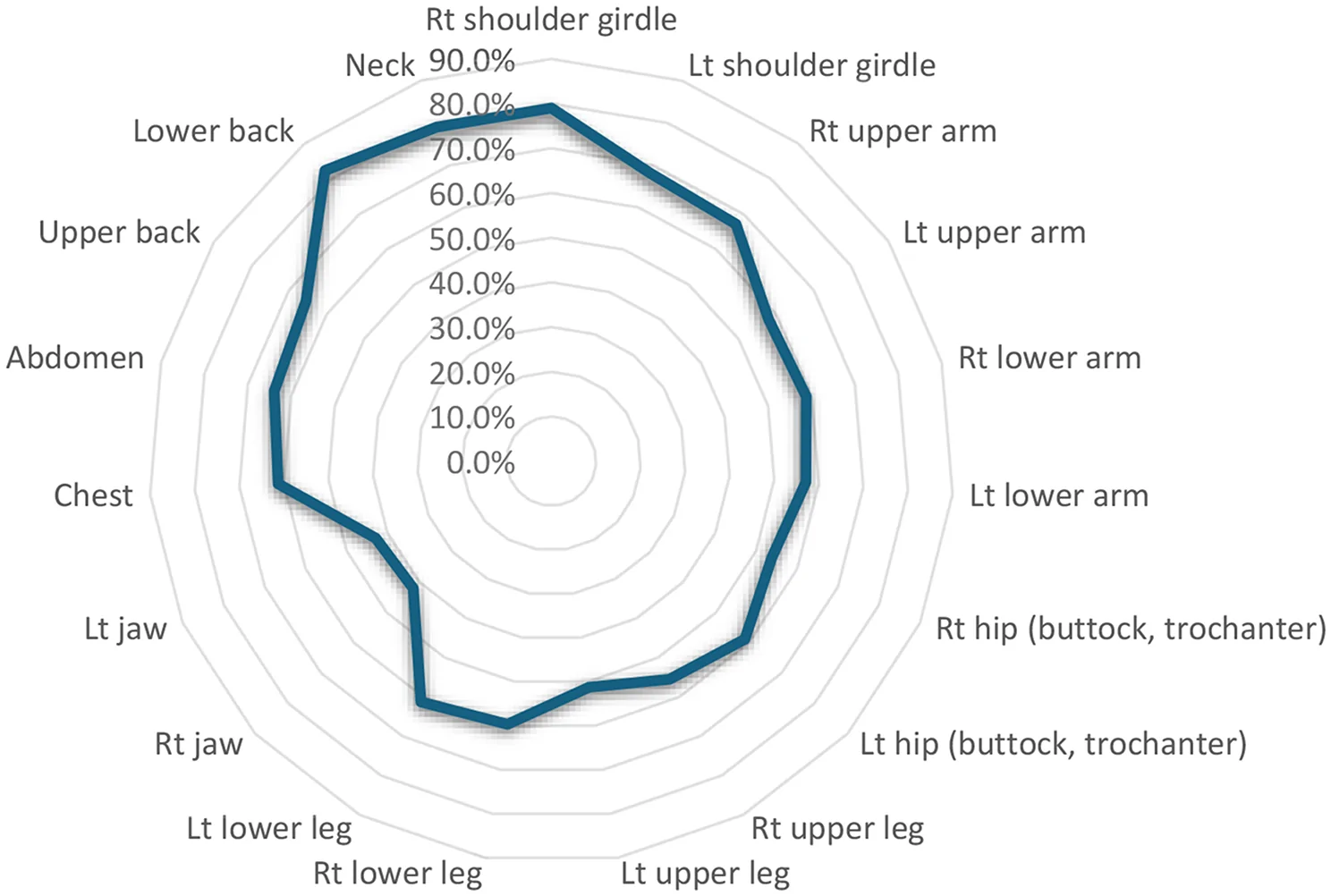
Sites of distribution of widespread pain index in SLE patients with fibromyalgia (*n* = 119)

**Fig. 3:**
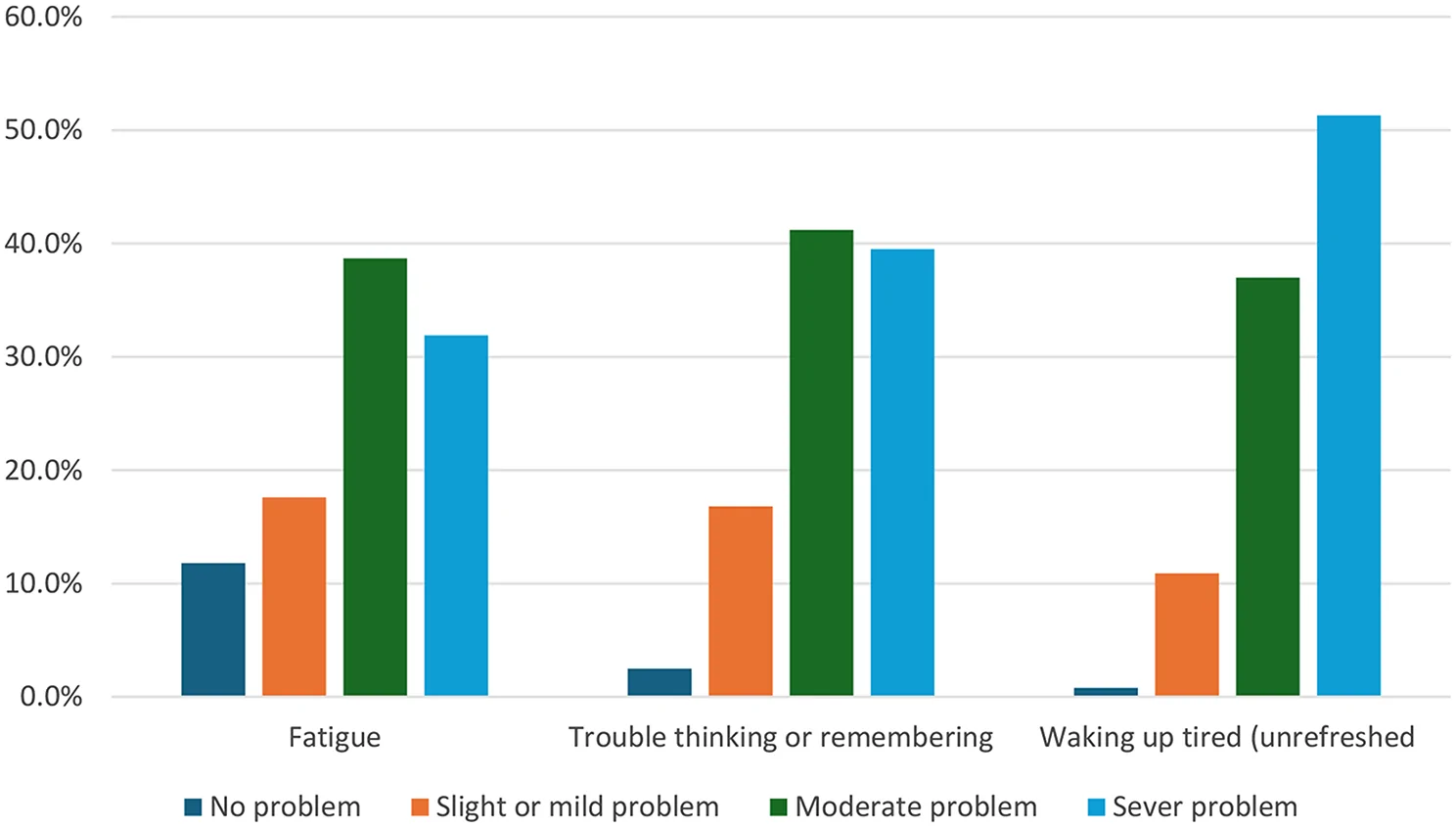
Symptom severity scale in SLE patients with fibromyalgia (*n* = 119)

Figure 2 depicts the sites of widespread pain distribution in the FM group. Based on body areas, the majority of FM patients experience pain in the lower back (82.4%), neck (79%), right shoulder girdle (79%), and right upper arm (67.2%), followed by the upper back (65.5%), abdomen (63.9%), and chest (61.3%). Figure 3 illustrates the SSS among FM patients, highlighting the severity of fatigue, trouble thinking, and waking up tired.

Table 3 illustrates statistically significant differences in sleep patterns between individuals with and without FM. The average sleep hours were markedly reduced in the FM group (*p* < 0.001). Other sleep disorders, such as snoring, sleep apnea, and the use of sleep medications, were more prevalent among FM patients.

**Table 3:** Sleep data of the study SLE patients (*n* = 550)

Variable mean ± SD, *n* (%), median(min.-max.)	Total (*n* = 550)	Non-fibromyalgia group (*n* = 431)	Fibromyalgia group (*n* = 119)	*P*
Sleep time				
Before 12 AM After 12 AM	252 (45.8 298 (54.2	222 (51.5) 209 (48.5)	30 (25.2) 89 (74.8)	**< 0.001**
Average hours of sleep per day	6.84 ± 1.71	7.00 ± 1.58	6.29 ± 2.03	**< 0.001**
Frequently wake up during sleep	306 (55.6)	201 (46.6)	105 (88.2)	**< 0.001**
Take naps during the day	186 (33.8	151 (35.0)	35 (29.4)	0.251
Take sleeping pills	74 (13.5)	45 (10.4	29 (24.4)	**< 0.001**
Snore during sleep	67 (12.2)	42 (9.7)	25 (21.0)	**0.001**
Sleep apnea or other sleeping disorders	118 (21.5)	69 (16.0)	49 (41.2)	**< 0.001**
Sleeping pattern changes during vacation or weekends				
No change Sleep earlier Sleep later	292 (53.1) 39 (7.1) 219 (39.8)	248 (57.5) 31 (7.2) 152 (35.3)	44 (37.0) 8 (6.7) 67 (56.3)	**< 0.001**

The HRQoL domain scores using SF 36 for the study SLE patients with and without FM are shown in Table 4. FM patients had statistically significantly lower scores across all domains.

**Table 4:** Health related quality of life using SF 36 among SLE patients with and without fibromyalgia

Variable mean ± SD, *n* (%), median(min.-max.)	Total (*n* = 550)	Non-fibromyalgia group (*n* = 431)	Fibromyalgia group (*n* = 119)	*P*	Effect Size (d)	Interpretation
Physical functioning	55 (0-100)	60 (0-100)	45 (0-100)	**< 0.001**	0.44	Small-Moderate
Role limitations due to physical health	0 (0-100)	25 (0-100)	0 (0-100)	**< 0.001**	0.91	Large
Role limitations due to emotional problems	0 (0-100)	33.33 (0-100)	0 (0-100)	**< 0.001**	0.98	Large
Energy/fatigue	40 (0-100)	40 (0-100)	25 (0–55)	**< 0.001**	0.92	Large
Emotional well-being	48 (0-100)	52 (0-100)	32 (0–84)	**< 0.001**	1.06	Large
Social functioning	50 (0-100)	62.50 (0-100)	25 (0-100)	**< 0.001**	1.05	Large
Pain	45 (0-100)	47.50 (0-100)	32 (0–80)	**< 0.001**	1.28	Large
General health	40 (0–95)	45.00 (0–95)	30 (0–80)	**< 0.001**	0.84	Large
SF-36 PCS	40 (0-96.25)	46.25 (3.75–96.25)	27.50 (0-64.38)	**< 0.001**	1.13	Large
SF-36 MCS	38.88 (0-100)	47.21 (1-100)	22.25 (0-73.38)	**< 0.001**	1.20	Large

MCS: mental component summary; PCS: physical component summary; SF-36: short form 36

Table 5 presents the univariate and multivariate logistic regression analysis. Independent predictors of FM in the multivariate model included age at SLE diagnosis (OR = 1.075, *p* = 0.008), severe SLEDAI score (OR = 0.396, *p* = 0.007), irritable bowel syndrome (OR = 0.339, *p* < 0.001), headache (OR = 0.356, *p* = 0.010), anxious mood (OR = 0.405, *p* = 0.033), low back pain (OR = 0.169, *p* < 0.001), and methotrexate use (OR = 2.723, *p* = 0.006).

**Table 5:** Univariate and multivariate logistic regression analysis of factors associated with fibromyalgia in SLE patients (*n* = 550)

Variable	Univariate Analysis			Multivariate Analysis		
	OR	95% CI	*p*-value	OR	95% CI	*p*-value
Age	1.034	1.013–1.056	**0.001**	0.965	0.916–1.016	0.179
BMI	1.045	1.009–1.083	**0.015**	0.736	0.942–1.043	0.991
Living alone	4.282	1.899–9.655	**< 0.001**	0.290	0.099-0.848	0.024
Low physical activity level	2.919	1.902–4.478	**< 0.001**	0.834	0.473–1.472	0.531
Age at SLE diagnosis	1.045	1.022–1.069	**< 0.001**	1.075	1.019–1.134	**0.008**
Sever SLEDAI score	4.126	2.513–6.774	**< 0.001**	0.396	0.202-0.776	**0.007**
Chronic kidney disease	1.764	1.070–2.909	**0.026**	0.854	0.410–1.780	0.674
Chronic hemodialysis	2.517	1.004–6.307	**0.049**	0.337	0.092–1.231	0.100
Chronic psychiatric disease	0.433	0.229–0.818	**0.010**	0.696	0.299–1.624	0.402
Chronic heart disease	2.176	1.067–4.438	**0.033**	1.919	0.666–5.529	0.227
Chronic lung disease	3.281	1.751–6.146	**< 0.001**	0.452	0.180–1.136	0.091
Hypertension	2.075	1.341–3.210	**0.001**	0.934	0.476–1.836	0.844
Diabetes mellitus	1.843	1.010–3.365	**0.046**	1.140	0.486–2.672	0.764
Fatigue	4.411	2.784–6.990	**< 0.001**	0.947	0.513–1.748	0.862
Irritable bowel syndrome	5.245	3.203–8.588	**< 0.001**	0.339	0.187–0.613	**< 0.001**
Amnesia	5.161	3.080–8.649	**< 0.001**	0.997	0.497–1.998	0.992
Headache	7.525	3.932–14.398	**< 0.001**	0.356	0.162–0.782	**0.010**
Anxious mood	9.504	4.970-18.177	**< 0.001**	0.405	0.176–0.929	**0.033**
Depressed mood	4.264	2.766–6.572	**< 0.001**	0.964	0.524–1.771	0.905
Low back pain	12.519	6.819–22.982	**< 0.001**	0.169	0.083–0.341	**< 0.001**
Methotrexate use	2.398	1.407–4.088	**0.001**	2.723	1.334–5.558	**0.006**

Bold P:<0.05

## Discussion

Although the prevalence of FM in SLE has been reported in various populations worldwide, there is a paucity of data from Egypt. This study addresses this gap by examining FM among Egyptian SLE patients using the 2016 revisions to the 2010/2011 FMS diagnostic criteria [18]. The study includes 550 SLE patients from multiple tertiary rheumatology centers and provides data about the relationship between FM and HRQoL among SLE patients.

In SLE patients, the prevalence of FM is significantly higher than in the general population [6], exhibiting a wide prevalence range of 1–61% [20]. Our findings indicated that 21.6% of the study SLE patients met the diagnostic criteria for FM. A recent meta-analysis of fifty-six studies involving 58,052 SLE patients revealed an overall random-effects pooled prevalence of FM at 15.8% [6]. In another study conducted in the United States, using survey criteria for FM, it was possible to identify FM in 22.1% of patients with SLE [21]. In a cross-sectional data analysis of the Spanish Registry comprising 3,591 SLE patients The prevalence of FM in the included SLE patients was 6.9%, depending upon the duration of the SLE disease [22].

The results of the current study revealed that FM group had a statistically significantly higher age and longer disease duration than non-FM group. This is in line with the results revealed by V. Torrente-Segarra and coauthors [22]. The severity of FM symptoms varies by age group, with younger and middle-aged patients experiencing worse FM symptoms than older patients [23]. Also, FM was more common in SLE patients with a disease duration of more than five years than in those with a disease duration of five years or less, according to a large registry involving patients with SLE. Patients with SLE duration > 5 years had a prevalence of 6.9%, while those with SLE duration ≤ 5 years had a prevalence of 4.0% (*p* < 0.05) [22]. However, in a study from India conducted on 158 SLE patients, the authors found that factors such as patient age and disease duration were similar in FM and non-FM patients [24].

There is increasing evidence indicating a correlation between a high BMI and pain, as well as painful disorders such as FM [25]. In the present study, BMI was statistically significantly higher in FM group. In fact, overweight and obesity are prevalent in FM [26]. In an Internet-based study, the results confirmed the coexistence of FM and a high BMI [27]. Increased BMI has been also associated with increased severity of FM and the prevalence of musculoskeletal pain in the general population [26]. The intricate connections between obesity and pain may be influenced by mechanical overload and several proinflammatory and neurohormonal pathways [28].

One of the most compelling issues is whether FM is independently associated with SLE activity, as has often been proposed. Nevertheless, certain studies have dismissed this possibility [10, 29, 30]. However, in the current study, we found that FM patients had higher SLEDAI scores. In fact, several SLEDAI components that were elevated in the FM group, such as headache and cognitive symptoms, rely heavily on patient-reported outcomes and may be influenced by symptom amplification inherent to FM. Future research should focus on elucidating the underlying mechanisms that may contribute to this association.

Chronic kidney disease (CKD) is a systemic condition that may also manifest musculoskeletal symptoms [31]. Additionally, more than 90% of hemodialysis patients experience musculoskeletal manifestations [32].According to the current study, the FM group had a higher prevalence of CKD and chronic HD. Although some studies suggest that FM prevalence among HD patients is comparable to that of the general population [33, 34], other studies indicate a higher prevalence of FM in this group [35, 36].

In the present cohort, MTX use was significantly more frequent among SLE patients with concomitant FM. Although MTX-related fatigue has been reported in inflammatory rheumatic diseases, particularly as a temporally related post-dose symptom, evidence remains insufficient to conclude that the higher fatigue burden in our FM group was MTX-induced [37, 38]. Conversely, fatigue in SLE is widely recognized as multifactorial and is often more strongly associated with FM, pain, poor sleep, stress, and depression than with lupus disease activity itself. Several studies have shown that fatigue and SLE activity follow distinct trajectories over time, and that FM may confound symptom interpretation and treatment response assessment [39–41]. Therefore, the greater frequency of MTX use in our FM group may reflect confounding by indication, with MTX being prescribed more often to patients with greater musculoskeletal symptom burden or perceived disease activity, rather than a direct causal role of MTX in FM-related fatigue [42].”

In general, the sleep quality of patients with SLE is worse than that of the general population [43].

Also, FM is thought to be a neurological condition brought on by an aberrant way that pain is processed. The disease is caused by a combination of factors, including physical stress, endocrine abnormalities, sleep disorders, central sensitization, heredity, and psychological factors [44].

About 90% of patients report having poor sleep, which is regarded as a fundamental symptom in the diagnosis of FM along with pain and fatigue. Fatigue, depression, pain catastrophizing, and other physical, emotional, and cognitive aspects of FM are all adversely affected by poor sleep quality [45]. In our cohort, the results revealed that the average sleep hours were significantly lower in the FM group and other sleep disorders, such as snoring and sleep apnea were more prevalent among FM patients. Over 80% of individuals with FM report significant fatigue or inadequate sleep quality, both of which are recognized by patients and healthcare professionals as primary therapeutic targets [46]. Understanding how FM patients manage poor sleep quality might help health practitioners tailor sleep education programs for this condition [47].

FM patients had statistically significantly lower scores across all domains in the present study.FM has been identified as a cause to work disability and diminished HRQoL in individuals with SLE, both cross-sectionally and longitudinally. FM serves as a main determinant of impaired physical and mental domains in SLE patients, correlating with increased fatigue [48]. A cross-sectional study involving 237 SLE patients indicates that FM severely impacts HRQoL, particularly in the domains of “pain” and “fatigue” [49]. The consequences of FM on HRQoL are considerably relevant, as they may lead to substantial challenges in executing daily living activities [50]. It was established by Offenbaecher et al. [51] that more than 50% of patients with FM claimed that their HRQ levels are not related to pain, but rather to other characteristics of FM.

The main strength of this study lies in its comprehensive approach to data collection and analysis, allowing for a nuanced understanding of the subject matter. Additionally, the diverse participant pool enhances the generalizability of the findings across different demographics.

Lastly, a few significant limitations should be taken into account. the reliance on self-reported data could introduce bias and impact the accuracy of the results. Additionally, sleep was assessed using a non-validated rather than a standardized tool such as the Pittsburgh Sleep Quality Index (PSQI), which limits the comprehensiveness of our sleep quality assessment. Furthermore, the study’s cross-sectional design limits the ability to establish causal relationships, making it difficult to draw definitive conclusions about the effects observed. Additionally, serological and immunological parameters, including ANA titers, anti-dsDNA antibodies, and complement levels (C3, C4), were not included in our analysis, which restricts our ability to fully characterize the immunological profile of the SLE cohort and explore potential associations between these parameters and FM. Another important limitation is the limited characterization of psychiatric comorbidities. Although the presence of physician-diagnosed chronic psychiatric disease was recorded, detailed information regarding psychiatric diagnoses, standardized diagnostic assessments, severity, duration, and treatment status was unavailable. Future research should aim to address these limitations by utilizing more diverse samples and employing longitudinal methods. Future longitudinal studies should also incorporate validated psychiatric screening instruments (such as the PHQ-9, GAD-7, or structured psychiatric interviews) to better define the independent contribution of psychiatric disorders to FM in patients with SLE. This approach would provide a more comprehensive understanding of the nature of FM among SLE patients. Moreover, integrating multiple data sources could enhance the validity of the findings and contribute to a more nuanced analysis of the underlying factors at play. Ultimately, this would pave the way for more informed interventions and policy decisions, allowing researchers and practitioners to better address the complexities of the FM.

## Conclusion

The findings suggest that FM is highly prevalent among Egyptian SLE patients and is associated with older age, higher BMI, longer disease duration, greater disease activity, disturbed sleep patterns, and significantly poorer HRQoL across all SF-36 domains. The strong association between FM and reduced HRQoL underscores the importance of screening for FM in SLE patients as part of routine clinical assessment. Future longitudinal studies are warranted to determine whether early identification and integrated management of FM in SLE patients could improve long-term health outcomes. Until such evidence is available, clinicians should remain vigilant for FM symptoms in SLE patients and adopt a holistic, patient-centered approach to address the multifaceted burden of these coexisting conditions.

## Data Availability

The datasets used and/or analysed during the current study are available from the corresponding author on reasonable request.

## Abbreviations

ANA: Antinuclear antibodies
BMI: Body mass index
BP: Bodily pain
CKD: Chronic kidney disease
EW: Emotional well-being
FM: Fibromyalgia
GHP: General health perception
HRQoL: Health-related quality of life
LBP: Low back pain
MCS: Mental component summary
MSK: Musculoskeletal
PCS: Physical component summary
PF: Physical function
RFE: Role function–emotional
RFP: Role function–physical
SF: Social function
SF-36: Short-Form Health Survey-36
SLE: Systemic lupus erythematosus
SLEDAI: SLE disease activity index
SLICC: Systemic Lupus International Collaborating Clinics
SSS: Symptom severity score
VT: Vitality
WPI: Widespread pain index
